# Making a murderer: Media renderings of brain injury and Aaron Hernandez as a medical and sporting subject

**DOI:** 10.1016/j.socscimed.2019.112598

**Published:** 2020-01

**Authors:** Hollin Gregory

**Affiliations:** School of Sociology and Social Policy, University of Leeds, LS2 9JT, UK

**Keywords:** Chronic traumatic encephalopathy, Crime, Concussion, Dementia, Media discourse, Neuroscience, Sport, Subjectivity

## Abstract

This paper examines the entanglement of medicine, brain injury, and subjectivity within newspaper discourse and through the case of ex-American footballer Aaron Hernandez. In 2017, two years after being found guilty of murder and five years after scoring in the Super Bowl, Aaron Hernandez died by suicide in his prison cell. Hernandez was posthumously diagnosed with Chronic Traumatic Encephalopathy (CTE), a neurodegenerative disease associated with violence, depression, and dementia-like symptoms. I examine newspaper coverage of the Hernandez case, focusing upon the murder, arrests, conviction, suicide, and diagnosis of CTE in order to examine understandings of Hernandez's subjectivity. I make three conclusions: First, the disease is not mentioned prior to diagnosis with family instability, friendship groups, individual psychology, and the entitlement of celebrity foregrounded. Second, CTE *is* foregrounded after the diagnosis and is used to explain much of Hernandez's behaviour. Third, the diagnosis of CTE goes someway to normalizing the behaviour of Hernandez, rendering his behaviours comprehensible. I conclude by considering how the specific narrative of CTE-as-acquired-dementia shapes depictions of Hernandez's subjectivity and discuss how this case troubles existing literatures on the neurologization of selfhood.

## Highlights

• CTE is a neurodegenerative disease popularly associated with contact sport.
• Aaron Hernandez was convicted of murder, died by suicide, and diagnosed with CTE.
• Through media analysis, I ask if Hernandez's actions were seen as shaped by CTE.
• CTE is not mentioned prior to suicide and rarely prior to formal diagnosis.
• After diagnosis CTE is used to explain and normalize much of Hernandez's behaviour.

## 1 Introduction

At 3am on April 19th, 2017 ex-American Footballer Aaron Hernandez was found dead in his prison cell having apparently hanged himself. Hernandez's death came just two years after a conviction for first-degree murder and five years since he scored a touchdown in the 2012 Superbowl for the New England Patriots. If Hernandez's path from sporting glory, to celebrity, to violence, and finally self-inflicted death was believed by many to be a profoundly American story (e.g. Patterson et al., 2018), what happened next was perceived as a harbinger of an American future: Hernandez's brain was removed from his body and sent to Boston University's Chronic Traumatic Encephalopathy (CTE) Center. Upon investigation it was discovered that Hernandez was suffering from an advanced case of CTE, a neurodegenerative disease associated with aggression, depression, and symptoms similar to Alzheimer's disease. CTE is believed to be caused by repetitive mild traumatic brain injury, quite possibly suffered on the football field. Hernandez's American story of sport, celebrity, and violence had become enmeshed with biology, medicine, and the brain.

This article is about that enmeshment. I use the Hernandez case to examine the ways in which neuroscience in general, and CTE in particular, shape articulations of subjectivity within the contemporary news media. From this analysis I draw three conclusions. First, and contrary to prominent theses which suggest that medicine and neuroscience have been centralized within contemporary discourse, media outlets largely did *not* discuss the possibility of CTE prior to Hernandez's posthumous diagnosis. Second, diagnosis itself *did* significantly affect media depictions of Hernandez, his crimes, and death by, third, rendering Hernandez as less monstrous. Hernandez was no longer depicted as totally Other but was instead brought into contact with others who play football or experience other forms of dementia.

## 2 Literature review

### 2.1 Chronic Traumatic Encephalopathy

Chronic Traumatic Encephalopathy, or CTE, is a neurodegenerative disease

‘ … clinically associated with symptoms of irritability, impulsivity, aggression, depression, short-term memory loss and heightened suicidality … With advancing disease, more severe neurological changes develop that include dementia, gait and speech abnormalities and parkinsonism.’ (McKee et al., 2013, p. 44, p. 44)

CTE is associated with a single significant risk-factor: repetitive mild traumatic head injury. It is frequently and plausibly argued that a prominent group at-risk of CTE are those who play contact sports: high profile case studies (e.g. Omalu et al., 2005) and epidemiological work (e.g. Bieniek et al., 2019) has examined the presence of the disease in samples consisting of such individuals. Nonetheless, a host of uncertainties remain and it continues to be argued that CTE is yet to be fully defined; that prevalence is unknown; and that diagnostic criteria are preliminary (Stewart et al., 2019, p. 231). Indeed, the most recent iteration of the high-profile ‘consensus statement’ on concussion in sport states that: ‘A cause-and-effect relationship has not yet been demonstrated between CTE and SRCs [Sports Related Concussions] or exposure to contact sports.’ (McCrory et al., 2017, p. 844).

Despite these uncertainties, a link has been suggested between boxing and brain injury since at least the 1920s (e.g. Martland, 1928). Concern regarding brain injury and other contact sports, however, began to manifest in the 1970s (Casper, 2018, p. 798). Indeed, an apparently decisive moment in the history of CTE came as recently as 2005 with the publication of the paper ‘Chronic Traumatic Encephalopathy in a National Football League Player’. Based around the autopsy of a brain belonging to Mike Webster, a hall of fame centre who played in the National Football League (NFL) for 16 years, the paper was the first to show ‘neuropathological changes consistent with long-term repetitive concussive brain injury’ (Omalu et al., 2005, p. 128). The story of this 2005 paper, and the apparent attempts of the NFL to cover up its findings, have been widely told, most prominently in the acclaimed film and book *League of Denial* (Fainaru-Wada and Fainaru, 2013) and the film *Concussion* (Landesman, 2015), starring Will Smith as lead author Bennett Omalu (see Furness, 2016 and; Ventresca, 2019 for analyses).

The “post-Webster” era has heralded what many commentators insist is a new moment for American football wherein medical and neuroscientific findings radically shape sporting practice. *League of Denial* itself proclaims the game to be under ‘existential threat’ from CTE (Fainaru-Wada and Fainaru, 2013, p. 6). Kevin McFarland of *The A.V. Club*, reviewing *League of Denial*, insists football ‘ … is a dead sport walking in the United States’ (2013). David Remnick, editor of the *New Yorker*, recently suggested that we had arrived at ‘football's long eclipse’ (2018).

The NFL itself has been accused of being slow to respond to – or even acknowledge – these challenges and, as the title suggests, *League of Denial* argues there have been deliberate attempts at obfuscation from within the league (Fainaru-Wada and Fainaru, 2013; Furness, 2016). Within this context, the aforementioned foregrounding of scientific uncertainties over the relationship between contact sport and CTE is itself subject to continued, rigorous critique (Casper et al., 2019; Finkel et al., 2019). The NFL have, meanwhile, implemented various rule changes apparently intended to prevent concussion (Knoblauch, 2018) and have increased the resources available to former players suffering with Alzheimer's and related health conditions (Fainaru-Wada and Fainaru, 2013, p. 214).

A small body of social scientific literature has considered understandings of brain injury within this changing landscape. First, there is a widely articulated belief that there has been a medicalization of concussion in recent years, with medical professionals playing an increasingly prominent role in the diagnosis of, and subsequent response to, concussions which occur in sporting contexts (e.g. Hardes, 2017; Malcolm, 2016). Second, there is a claim – often substantiated through media analysis – that the medicalization of concussion is deeply entwined with changing modes of masculinity and subjectivity. It has long been argued that sport is a key locus in the perpetuation of contemporary forms of ‘hegemonic masculinity’ (Connell, 2005) and, furthermore, that ‘bodies [are used in sport] as weapons in order to attain cultural norms and ideals concerning masculinity’ (McGannon et al., 2013, p.395). While there is a widespread recognition that significant injury may follow the use of the body as a weapon, there has been an apparently widespread ‘informed soldier’ (Furness 2016 p.51) narrative which suggests that athletes knew and accepted the bodily risks they took in pursuit of sporting glory and masculine ideals. It has been argued, however, that the interjection of a CTE-based discourse is significantly changing the picture.

Anderson and Kian, for example, argue that increased awareness of CTE, combined with a more widespread softening of masculine ideals, has opened a ‘crack in the hegemonic system’ which is allowing space for non-hegemonic forms of masculinity to be expressed in sport (2012, p. 153; See also: Rugg, 2019, p. 58). Furness suggests that the possibility of a link between concussion and CTE actively undercuts the aforementioned informed soldier trope because while players accepted *bodily* injuries they did not accept the possibility of *brain* injuries about which they knew very little (2016, p. 52). Ventresca has examined the ‘multiple becomings’ of CTE within media discourse, arguing that CTE variously refers to ‘a medical diagnosis, an object of scientific study, a cultural phenomenon, and a lived experience’ (2019, p. 137). Ventresca argues, as does Furness, that many former athletes form an identity around the possibility and fear of CTE but also, and significantly, that the uncertainties associated with CTE as a medical diagnosis have been strategically used to undercut any possibility of action based upon such fears. Analyses conducted by Adam Rugg (2019) support this general thesis about the importance of CTE in sport while also suggesting that, as with other injuries (Haslerig et al., 2018), the way in which individual players are interpellated by discourses pertaining to CTE is significantly raced.

It is the claim advanced within this emerging body of literature – that CTE has become central to understandings of sporting cultures and sporting subjects – which is my concern here. In particular, I consider how the media depict sporting subjects when they are brought into the purview of a medical and neuroscientific gaze. In examining this topic, I draw upon, and contribute to, broader considerations of the ways in which biomedical knowledge affects understandings of ourselves, others, and society.

## 2.2 Biomedicine and neuroscience in the media

‘[N]euroscience is increasingly visible in the popular press’ (O'Connor and Joffe, 2013, p. 257) and, as is the case with other areas of biomedicine (Michelle, 2007), coverage has been largely positive and non-critical (Atteveldt, 2014, p. 9; Racine et al., 2010, p. 729). Perhaps surprisingly, there has been relatively little discussion of this increasingly prominent coverage of biomedicine from within journalism and media studies. Indeed, some scholars have suggested that the fields are complicit in the perpetuation of dominant scientific narratives (Hallin and Briggs, 2015). There is, however, a substantial literature within medical sociology and science and technology studies wherein sustained investigation of biomedicine has taken place. In particular, and of direct relevance to my present analysis, there have been considerations of how biomedical discourses affect conceptions of personhood.

Dumit, for example, has argued that ‘ … at least in the United States, expert scientific and medical *facts* play a key role in how we experience our selves, our bodies, and others’ (2004, p. 160, italics in original). Dumit calls this process ‘objective self-fashioning’ (2003, p. 39) and is not alone in suggesting that biomedicine is increasingly important within such processes. Rabinow suggested that with the emergence of novel biotechnologies, principally in genetics but also the neurosciences, we would see ‘the likely formation of new group and individual identities and practices arising out of these new [scientific] truths,’ a phenomenon Rabinow referred to as “biosociality” (1999, p. 413). Nikolas Rose has referred to such self-fashioning through neurological discourse as resulting in “neurochemical selves” (2003, 2004) while Vidal has opted for the notions of “brainhood” (contra personhood) and “cerebral selves” (Vidal, 2002, 2009; Vidal and Ortega, 2017). Various forms of media play an important role in these processes. Pickersgill and colleagues, for example, found that the mainstream media ‘relied upon and contributed to a wider cultural understanding of the brain as playing a role of (mundane) significance for people's sense of self’ (Pickersgill et al., 2017, pp. 98–99) and may advance a “neuro-essentialism” (Racine et al., 2010) which equates brain with self.

There are two particular consequences of this perceived turn towards biomedical understandings of personhood which are important to note in the context of media representations of brain injury. First, there has been an apparent reshaping of the contours of responsibility. If alcoholism, cancer, and criminal behaviour (to name commonly cited examples) are believed to have root in one's brain and biology, and therefore lie outside of one's control, then an individual may cease to be understood as morally culpable for their status or actions (Easter, 2012; Weiner, 2009). Simultaneously, meanwhile, new forms of biological knowledge may lead to novel responsibilities. Novas and Rose, for example, suggest that in context of Huntington's Disease, genetic knowledge comes with considerable responsibilities and affects ‘life-planning decisions concerning education, careers, relationships and children’ (2000, p. 505). Thus, an understanding of subjectivity which sees traits and behaviours as shared amongst a group united by their biology may result in an individual who understands themselves as having a series of *new* obligations and responsibilities (Biebricher, 2011; Dimond, 2014; Hallowell, 1999).

Second, it has been argued that if individuals are equated with brain states there will be an increasing demand to govern and monitor those with “risky brains” (Rose, 2010). In this context, the brain becomes an ‘index of difference’ (O'Connor et al., 2012, p. 225): “normal” individuals cease to see those with risky brains as being like themselves and, instead, construct them as radically Other:

‘On the one hand there are fantasies of security, imagined communities where normal individuals and families can live an untroubled life of freedom. And, as its inescapable reciprocal, there is a constant fear of predatory monsters [Rose gives imagined sex offenders, paedophiles, serial killers, and ‘deranged mental patients freed to kill again’ as examples] … A monstrous individual is an anomaly, an exception. This is not merely one who diverges from a norm, but one who is of a radically different nature, implacably pathological, evil.’ (Rose, 2010, pp. 87–88)

This literature on neuroscience and personhood, first, provides an important background against which to understand the “cultural phenomenon” of CTE and, second, reaffirms the need to meaningfully engage with the ways in which emerging biological and neurological knowledge are articulated in media contexts.

There are, nonetheless, reasons for caution when making grand claims about the bio- or neuro-logicalization of personhood. Examining media coverage of neuroscience, for example, O'Connor and Joffe dispute the claim that the emergence of neuroscience has led to an overhaul of longstanding concepts of responsibility and self-control, arguing that there have been only ‘superficial reframings’ of the matter (2013, p. 225). Elsewhere, O'Connor et al. argue that neuroscience is ‘assimilated in ways which perpetuate rather than challenge existing modes of understanding self, others and society’ (2012, p. 225). Just as significantly, research has increasingly queried the extent to which diverse subjects draw upon biomedical knowledge *at all* (Pickersgill et al., 2011; Weiner, 2011; Weiner et al., 2017).

A crucial methodological point is relevant to this apparent critique. Within a significant portion of the existing literature, a moment is pre-selected where it is known in advance that neuroscience is highly relevant (by, for example, searching all newspaper articles with the word “neuroscience” in the title). These studies are crucial in understanding neuroscience as a practice and the brain as a particular object. If, however, the intention is to examine the diffusion of medicine/neuroscience/the brain through society, the approach risks ‘chasing its own tail, offering up its own agendas and categories and getting those same agendas and categories back in a refined or filtered or inverted form’ (Potter and Hepburn, 2005, p. 293). In other words, it may be misleading to select a moment when it is known in advance that subjectivity and the brain are entwined (or to artificially create such an entwinement within an interview) and, upon finding entwinement, to exclaim that the phenomena is found everywhere and anywhere.

Such a conclusion is applicable to many studies examining concussion in the media, for many studies focus their analysis upon articles pre-selected because they are known to consider brain injury: either through analyses of how the media responded to a particular high-profile concussion (Anderson and Kian, 2012; McGannon et al., 2013); or through a wider analysis of how brain injury is covered in the media (Ahmed and Hall, 2017; Furness, 2016; Ventresca, 2019). These studies are crucial in understanding the construction of concussion *as a diagnosis* and have aptly demonstrated that particular individuals have been interpellated by a discourse informed by CTE. These pieces help us to understand the ways in which concussion is understood and reported upon. By preselecting a moment when it is already known that head trauma is relevant, however, these studies do not readily facilitate an examination of concussion/CTE's *broader resonance* within sport and society for they cannot examine, for example, when or if a discourse related to CTE is *absent*. Any claim that CTE has permeated culture more broadly, that it provides a general discourse through which to understand players' actions or subjectivities, thus necessitates an additional form of analysis.

## 2.3 Aaron Hernandez

The case of Aaron Hernandez allows us to examine if and how concussion and CTE shape understandings of sporting subjectivity more broadly. Hernandez was an All-American athlete throughout high school and college, setting individual records and winning a collegiate national championship while playing for the Florida Gators in 2009 (Hernandez and Andersen, 2018, p. 69). In 2010, Hernandez became the youngest player in the NFL when he was drafted by the New England Patriots (Patterson et al., 2018, p. 115). Hernandez achieved a huge degree of success, scoring a touchdown in the 2012 Superbowl and subsequently signing a 5-year, $40 million contact which was one of the highest ever awarded to a tight end in the NFL (Patterson et al., 2018, p. 152).

The current analysis, however, is centred on the following events. On June 17th^,^ 2013 a man named Odin Lloyd, who was in a relationship with the sister of Aaron Hernandez's fiancée, was found murdered after suffering multiple gunshot wounds: one week later, on June 26th, Hernandez was arrested for the murder. Just under a year later, on May 15th^,^ 2014 and while in prison, Hernandez was charged with two further ‘drive-by’ murders, those of Safiro Fertado and Daniel de Abreu. On April 15th^,^ 2015, Hernandez was convicted of the murder of Lloyd and sentenced to life imprisonment without the possibility of parole.

In April 2017 came a series of events in quick succession: On April 14th^,^ 2017 Hernandez was found not guilty of the murders of Fertado and de Abreu. On April 19th, Hernandez was found dead having apparently hanged himself in his cell. His family promptly sent his brain to Boston University to be studied for signs of CTE. Several months later, on September 21st^,^ 2017, a press release from Boston University confirmed Hernandez was suffering from a severe case of CTE.

What is methodologically useful about the above is the following. First, all the incidents – from the murder of Odin Lloyd to the posthumous diagnosis of CTE – happen in the “post-Webster era”, well into the time of “CTE as cultural phenomenon”. Second, given Hernandez's posthumous diagnosis, we know that the media will ultimately deem neurodegenerative disease as relevant to understanding Hernandez and his actions. Crucially, however, Hernandez's diagnosis of CTE arrives *after* the events in question had received significant media attention. These developments mean that finally, third, we have the opportunity to examine if and how CTE enters media discourse *prior* to his diagnosis and thus see just how deeply the discourse runs through contemporary media discourse.

## 3 Method

### 3.1 Sample: newspapers

I gathered articles from a cross section of US newspapers with a sample size significant enough to allow for meaningful patterns to be made evident. Outlets were considered which had a large circulation in the US and, in toto, were geographically dispersed. Ultimately, six outlets were selected for inclusion: *The Boston Globe* (BG), *The New York Post* (NYP), *The New York Times* (*NYT*), *USA Today* (USAT), *The Wall Street Journal* (WSJ), and *The Washington Post* (WP).

Content from these outlets was collected via the use of various searchable databases. The BG was searched through *Factiva*; NYP, *NYT*, USAT, and WP were searched through *Nexis*; and WSJ was searched through *ProQuest*.^1^ In all cases the search string “Aaron Hernandez” was used to identify applicable articles. To remove duplicates, *Nexis* was searched on the “moderate” and *Factiva* on the “similar” duplicate setting. Any additional complete or near-complete duplicate articles which escaped filters and became apparent during analysis were also removed. The decision was made to focus exclusively upon print editions for the sake of completion and consistency. No additional selection criteria needed to be met in order for an article to be considered.

### 3.2 Sample: dates

Six key events were initially identified for analysis:

• The murder of Odin Lloyd.
• The arrest of Aaron Hernandez for the murder of Lloyd.
• The arrest of Hernandez for the murder of Daniel de Abreu and Safiro Furtado.
• The conviction of Hernandez for the murder of Lloyd.
• The suicide of Hernandez.
• The announcement that Hernandez had CTE.

**Fig. 1:**
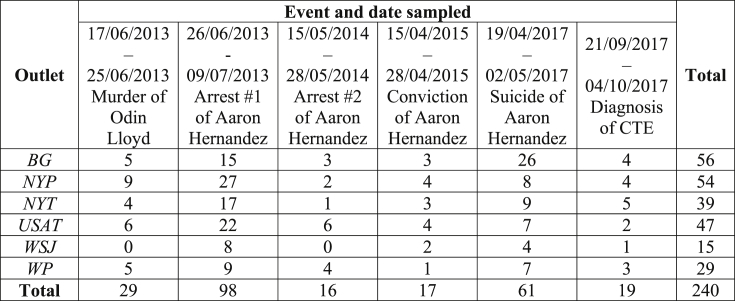
Details of newspaper articles sampled and the events to which sampling corresponds. The first date in each sampling period corresponds to the date on which the event in question occurred (i.e. Odin Lloyd was murdered on the 17th of June, 2013).

All articles published within two weeks of each event^2^ and which included the search string “Aaron Hernandez” were included in analysis. Coverage of these six events yielded a total sample of 240 pieces. A breakdown of publication dates and venues is provided in Fig. 1.

### 3.3 Analytic framework

Atteveldt, 2014 have previously noted ‘timing, topic, and newspaper type’ is of relevance to media reporting of neuroscience. The primary goal of this paper is to examine changes in narrative over time rather than to examine differences between publications. While I note, for example, that the BG pays greater attention to prison suicides than other outlets, my primary focus is upon how narratives shift as the focus moves from, e.g., suicide to the diagnosis of CTE.

I focus in particular on the construction of Hernandez as a subject within media discourse. There is a particular attentiveness to questions of causality (why did what happened happen?), culpability (who or what is responsible for what happened?), and kinship (who is Hernandez like?). Answering these questions involved interweaving two forms of analysis. First, a general inductive analysis (Thomas, 2006) aimed at discerning re-occurring themes within the sample. As an example, one prominent theme was the “Death of Dennis Hernandez” (Aaron Hernandez's father): this theme suggests that the death of a parent was key to understanding causation and Aaron's turn to crime. Second, a Foucauldian discourse analysis akin to that outlined by Parker (1999). Here the intent is not to identify themes but, rather, to examine how media texts ‘form the objects of which they speak’ (Foucault, 2002, p. 54). In order to do this, I itemized the subjects (e.g. Aaron Hernandez, the NFL, Odin Lloyd), objects (e.g. brain, cars), and places (e.g. houses, prisons) within the text and noted the terms with which they are associated. So, for example, Aaron Hernandez is frequently described as “emotionless” and “cold” and this, it seems, tells us something about the type of subject who is taken to commit crimes like these.

The aforementioned combination of thematic and Foucauldian discourse analysis is well placed to examine questions of causality, culpability, and kinship in the case of Hernandez by allowing an inquiry into which topics are raised when (thematic analysis) and, once that has happened, how key subjects and objects are constructed (Foucauldian discourse analysis). To be clear, I am not analyzing the accuracy of media discourse. It is not my concern here whether Aaron Hernandez really killed Daniel de Abreu and Safiro Furtado or whether CTE explains his actions. These are self-evidently important questions, but they will not be addressed here.

## 4 Analysis

### 4.1 Murder of Odin Lloyd

In the week between the murder of Odin Lloyd and the arrest of Aaron Hernandez a prominent theme was that this story was a familiar one (*NYT*, June 21, 2013) and that Hernandez, as a high-profile athlete caught up in a criminal investigation, ‘has a lot of company’ (*USAT*, June 21, 2013#2). “Company,” in this case refers exclusively to other NFL players who have been investigated by the police (*USAT*, June 24, 2013; *USAT*, June 25, 2013). Discussed in this context is OJ Simpson although in the case of Simpson it is both criminal and aesthetic similarities which capture the attention. The ‘media swarm’ around Hernandez's house and the ‘mundane pursuit’ of Hernandez as he drove a white SUV to the New England Patriots' stadium – while being filmed via helicopters – are both taken to evoke Simpson's famous police chase which occurred, almost to the day, 19 years prior (*NYT*, June 21, 2013).

Hernandez himself is described as ‘hot-tempered’ (*NYP*, June 22, 2013#2) and there are ‘red flags’ and ‘worrying signs’ in his past, a failed drug test being a prime example (*USAT*, June 21, 2013#2). There are also suggestions of ‘possible gang ties’ (*USAT*, June 21, 2013#2); questions over ‘the company he keeps’ (*BG*, 22 June, 213#1); and possible involvement in another shooting (*NYP*, June 20, 2013#1; *USAT*, June 24, 2013; *WP*, June 21, 2013). While it is noted that Hernandez has never been suspended (*USAT*, June 21, 2013#2); is subject to misinformation (*BG*, 25 June, 2013#1; *NYT*, June 25, 2013; *USAT*, June 25, 2013); and has not been declared a suspect (*NYP*, June 19, 2013; *NYP*, June 22, 2013#2; *WP*, June 20, 2013), his actions still cause a significant degree of suspicion and the loss of endorsements and associated finances is noted (*BG*, 22 June, 2013#2; *WP*, June 22, 2013). Even prior to Hernandez's arrest, therefore, certain themes begin to emerge: his psychology, his friendship group, and his kinship with other (sometimes alleged) criminals in the NFL all draw attention.

### 4.2 Arrest of Aaron Hernandez for murder of Odin Lloyd

Straight after Hernandez's arrest, a number of themes begin to emerge. First, there is the question of his *appearance* – his ‘dumb-sullen jaw’ and ‘dead jailhouse gaze’ (*WP*, 3 July, 2013#1); muscled-up or ‘action hero’ arms (*BG*, 9 July, 2013#1); and finally his tattoos – which prove a cause of continual fascination (e.g. *NYP*, 28 June, 2013#1; *NYP*, 28 June, 2013#2).

Second there is the question of *precedent*. Into this category can be found numerous, lengthy descriptions of a bar fight Hernandez was involved in while at The University of Florida. The fact that Hernandez was in this bar with Tim Tebow – the ‘mild-mannered’ (*USAT*, 3 July, 2013#2) and ‘God fearing’ (*NYP*, 28 June, 2013#1) quarterback who was already ‘a fully-fledged phenomenon’ ‘beloved’ for his ‘strong faith’ and ‘motivating personality’ (*NYT*, 7 July, 2013#1) and who clearly stands in contra-distinction to Hernandez – gives the story extra purchase. As was the case prior to his arrest, there are also discussions regarding the possibility that Hernandez previously shot a friend (*BG*, 6 July, 2013; *NYT*, 28 June, 2013#2; *WSJ*, 27 June, 2013#1). The murders of Fertado and de Abreu, which are being linked to Hernandez for the first time, are also discussed (e.g. *BG*, 9 July, 2013#2; *NYP*, 29 June, 2013#1; *NYT*, 28 June, 2013#2).

Third, there is the matter of *psychology*. Hernandez is repeatedly described as ‘emotionless’ (*NYT*, 28 June, 2013#1; *USAT*, 27 June, 2013#2) or as displaying ‘little’ or ‘no emotion’ while in court (*NYP* 27 June, 2013#1; *NYP* 27 June, 2013#2; *NYT*, 27 June, 2013). Hernandez is variously ‘agitated’ (*NYT*, 27 June, 2013), ‘angry’ (*NYT*, 29 June, 2013#3; *WSJ*, 27 June, 2013#1), ‘furious’ (*NYP*, 27 June, 2013#2), ‘immature’ (*USAT*, 9 July, 2013), and has ‘low self-esteem’ (*BG*, 3 July, 2013#3). He is described as has having killed in a ‘cold-blooded fashion’ (*USAT*, 28 June, 2013#3) – perhaps even being the NFL's first ‘serial killer’ (*NYP*, 28 June, 2013#1; *NYP*, 30 June, 2013#1). In addition, there is a discussion in both the *BG* (4 July, 2013) and the *WSJ* (3 July, 2013#1; 3 July, 2013#3) of personality tests undertaken prior to the NFL draft which show his ‘low social maturity’ and that he ‘enjoys living on the edge’.

When it comes to theories of *causation* or *explanation* there are two dominant threads. The first might be referred to as “psychosocial” factors which pertain to the ‘rocky life’ (*WSJ*, 27 June, 2013) of Hernandez, particularly while growing up in his home town of Bristol, Connecticut. It is repeatedly noted that Hernandez's father died when he was a teenager (*NYP*, 28 June, 2013#1; *NYT*, 27 June, 2013; *NYT*, 29 June, 2013#3; *WSJ*, 27 June, 2013; *WP*, 3 July, 2013#2) and that this had a profound impact upon him. Sometimes woven into the same narrative is a discussion of the ‘rough-and-tumble’ (*NYT*, 27 June, 2013) crowds Hernandez hung out with in ‘hardscrabble’ Bristol (*NYT*, 29 June, 2013#3). This sometimes leads to questions about ‘thug associates’ (*NYP*; 9 July, 2013#2), ‘sordid characters’ (*BG*, 3 July, 2013#3), and ‘hanging around with gangbangers’ (*BG*, 5 July, 2013). Celebrity is sometimes seen to play into this matter, as in the suggestion in *USAT* that Hernandez was part of a ‘rich jock culture’ (27 June, 2013#3).

That there is a racialized dimension to these descriptions is rarely made explicit, although a piece in *NYP* blames ‘culture’ for Hernandez's acts before bemoaning the fact that ‘those in the highest places, those who could make the biggest differences — starting with our first black president - choose to pass on addressing such elephant-in-the-room matters’ (1 July, 2013#2). It is important to note, however, that much of the vocabulary used to describe Hernandez has been critiqued by media studies scholars. “Thug,” for example is as ‘ … a term that carries powerful negative connotations for African American males and is considered by many to be a code for the N-word’ (Page et al., 2016, p. 2) and that has been put into a lineage from the term “brute” (Smiley and Fakunle, 2016, p. 354). The term “gangbanger” has been similarly described (Lopez, 2015, p. 5). While Hernandez is of Italian and Puerto-Rican heritage (Hernandez and Andersen, 2018, p. 5), others have noted that these terms have been transposed onto Latino communities (Brown et al., 2018) and it is perhaps worthy of attention that Hernandez's co-defendants were African American, as was his fiancée, as was his victim, as are the 11 other NFL players who are in the ‘National Felons League’ (*NYP*, 4 July, 2013#2) and who are discussed within the media at this time (e.g. *NYT*,28 June, 2013#1; *USAT*, 1 July, 2013#2). Given that the coverage of Hernandez occurred in the context of a media ecology in which the talk (Page et al., 2016), bodies (Haslerig et al., 2018), and behaviour (Rugg, 2019) of athletes is consistently interpreted in raced terms, such a finding should not be surprising.

The second theme relating to causation might be called ‘institutional enablement’. Here the lens falls upon NFL, the New England Patriots, and the University of Florida, with the television broadcaster ESPN also coming under occasional criticism. This institutional collective is sometimes referred to as ‘Football Inc.’: Those individuals and organizations who ‘looked the other way’ or actively subverted attempts to intervene in a life ‘spiralling out of control’ (Hohler, 2018). The Florida Gators, for whom Aaron Hernandez played in college, had an ‘unsavoury underbelly’ with a significant number of player arrests (*NYT*, 7 July, 2013) – this high number of arrests came despite a local law firm providing pro bono work for players (*WSJ*, 9 July, 2013), thus ensuring that Hernandez was able to get ‘off the hook’ (*USAT*, 3 July, 2013#2). A piece in the *NYP* argues, ‘[Urban] Meyer [Hernandez's coach in Florida] clearly didn't give a rat's rectum about safety when he spent five years inviting and indulging dangerous, criminally inclined players to the campus of the University of Florida … ’ (5 July, 2013). The Patriots, meanwhile, repeatedly ‘took a chance’ on players like Hernandez (*NYT*, 29 June, 2013#1) and were left in a ‘pile of rubble’ (*NYP*, 30 June, 2013#1). The NFL have ‘been here before’ (*NYT*, 28 June, 2013#1) and appear to have ‘zero impact on [a player's] moral compass' (*BG*, 3 July, 2013#4).

In light of the suggestion that there has been a “cultural awakening” to CTE it is worthy of note that, across the 98 articles published after Hernandez's arrest, there was no mention of Hernandez's brain or neurodegenerative disease. Instead, Hernandez's individual psychosocial background, affiliates (who are understood in raced terms), and the institutional indulgence of powerful organizations are all foregrounded. This is strong evidence to suggest that, while CTE may well be a “cultural phenomenon” in the second decade of the twenty-first century, subjects are less readily interpellated into its discourse than they are for other, existing, explanations of behaviour.

### 4.3 Arrest of Aaron Hernandez for murder of Safiro Fertado and Daniel de Abreu

While the arrest of Hernandez for the double-murder of Safiro Fertado and Daniel de Abreu did not radically change the media discourse, there are some novel concerns within this corpus. The first concerns how Hernandez should be labelled now that he has been formally linked to multiple deaths. An article in *USAT*, entitled ‘Hernandez profile fits as gangster, not serial killer’ (16 May, 2014#2), discusses the possibility that Hernandez has ‘gang ties’. It is argued in this article that Hernandez acts like an ‘old school mobster’, adheres to a ‘street code’, and is not a ‘serial killer’: This is a judgement reached by a forensic psychiatrist on the basis that Hernandez has friends, emotions (rage, in particular), and a ‘personality structure’. These, it is argued, are not characteristics associated with serial killers. A similar theme is discussed in the *WP* (16 May, 2014#4) wherein it is asked if Hernandez is ‘a spree killer? A gang killer?’. Of greater concern within this piece in the *WP* is the argument that Hernandez is *not* like others in the NFL (contra one of the themes identified above). The explanation for Hernandez's acts lies ‘buried deep in him’, ‘a dramatic failure of an internal mechanism’. Hernandez is described in this article as ‘an extreme anomaly’ and ‘profoundly unnatural’ – this is evidenced by the fact that ‘even piranhas don't turn their teeth on each other’. While the sample is small, the formal linking of Hernandez to multiple deaths seems to distance Hernandez from others in the NFL who have committed criminal acts. Through this distancing, it is psychosocial causes “within” Hernandez which are emphasized, rather than CTE or another diagnosis.

### 4.4 Conviction of Aaron Hernandez

The media coverage following the conviction of Hernandez for the murder of Odin Lloyd is largely continuous with that after his arrest. There is a continued focus upon Hernandez's psychology – he remains ‘prone to anger’ (*BG*, 16 April, 2015#1) whilst, in court, being ‘impassive’ (*NYP*, 16 April, 2015#2) and showing little emotion (*NYP*, 16 April, 2015#3; *WSJ*, 16 April, 2015#1). Indeed, an article in the *BG* formalises these traits and diagnoses Hernandez as a ‘sociopath’ (*BG*, 16 April, 2015#1). Explanations for this psychology once again emphasize the death of Hernandez's father (*NYT*, 16 April, 2015#2; *USAT*, 16 April, 2015#3) and Hernandez's upbringing in the ‘hardscrabble’ town of Bristol – described as ‘flagging’ and ‘prowled by petty criminals’ in the *NYT* (16 April, 2015#2). Hernandez ‘never fully separated’ (*NYT*, 16 April, 2015#1) himself from this upbringing.

Institutional enablement is also returned to after Hernandez's conviction. Hernandez was ‘shielded from wrongdoing’ (*NYT*, 16 April, 2015#2), ‘not held accountable’ (*USAT*, 16 April, 2015#3), and treated with ‘wonderment’ and ‘reverence’ because of his athletic talents (*BG*, 16 April, 2015#1). This argument is made most clearly in the below quote:

‘But it's even more important [to remember that athletes need to ‘live within the law’] for the coaches, agents, hangers-on and, yes, even fans who feed these athletes' egos, enabling their boorish behavior. They might not have pulled the trigger, but they all had a hand in making Hernandez believe he could.’ (*USAT*, 16 April, 2015#3)

In this context it is perhaps unsurprising that Hernandez is, again, grouped together with other NFL players who committed criminal acts.

### 4.5 Suicide of Aaron Hernandez

In media content considered thus far there has been no mention of Hernandez's brain, CTE, or his biology beyond reference to his athletic talents. That situation more-or-less forcibly changes after Hernandez's suicide; this is so not only because Hernandez's family sent his brain to Boston University to be tested for CTE but also because this demand *itself* became a spectacle: When Boston's medical examiner refused to release Hernandez's brain promptly there was a ‘a battle for the brain’ (Baez, 2018, p. 235) which ended up with Jose Baez, Hernandez's attorney, giving an impromptu press conference alleging that the brain was being kept illegally by the state.

Nonetheless, reporting on CTE remained sparse: the *NYT* (21 April, 2017#1) published a reasonably lengthy piece noting that CTE was linked with aggression. That article also noted that several players who had previously played in the NFL had died by suicide and were then found to have CTE. The *NYP* (21 April, 2017) noted in passing that Hernandez's brain had been released and the *BG* reprinted the district attorney statements which included information about the brain (20 April, 2017#2; 21 April, 2017#3). There is next to nothing, however, which works this discussion of CTE explicitly through Hernandez's behaviour or subjectivity.

Instead of a decisive turn to the brain, two other topics are discussed at reasonable length. Firstly, the ‘swirling rumours’ (*BG*, 25 April, 2017#2) concerning Hernandez's sexuality. It is claimed that bisexuality was Hernandez's ‘most guarded secret’ (*NYP*, 22 April, 2017) and there is some discussion of a ‘prison beau’ who may have been an intended recipient of a suicide note (*NYP*, 25 April, 2017). The *BG* in particular also understands Hernandez in the context of prison suicides in Massachusetts, discussing at length the long-term failings of the state's prison system (19 April, 2017#3; 20 April, 2017#9; 20 April, 2017#10). Beyond these additions, the narrative stays largely the same: there are the same discussions about Hernandez's ego; his father's death; the ‘ne'er-do-wells’ (*NYT*, 20 April, 2017); and the fall which ‘started on the streets of Bristol’ (*NYP*, 20 April, 2017#2).

### 4.6 Diagnosis of CTE

Finally, it is necessary to turn to newspaper coverage in the days after Hernandez's posthumous diagnosis with CTE. There are several themes in this coverage which seem worthy of note: First, while there is significant discussion of the uncertainties associated with CTE – the fact that it can only be diagnosed posthumously (*NYT* 22 September, 2017; *NYT*, 23 September, 2017#2; *USAT* 4 October, 2017); that, because the sample of brains donated to brain banks is ‘small and nonrandom’ (*NYT* 23 September, 2017#1) there is the possibility of bias (*NYT*, 25 September, 2017; *WSJ*, 22 September, 2017); that drug use is a potentially confounding variable (*NYT* 25 September 2017) – there is little hesitation in applying CTE to football (*WSJ*, 22 September, 2017), violence, suicide (*NYT*, 23 September, 2017#1; *NYT*, 25 September, 2017), and Aaron Hernandez as an individual. It is a ‘natural presumption’ (*NYT*, 25 September, 2017) that CTE leads to murder-suicide and ‘may explain his prison suicide’ (*NYP*, 23 September, 2017). As one op-ed in the *NYT* states:

‘C.T.E. deprives such players of the ability to handle disputes rationally. Indeed, being afflicted with C.T.E. may well equate to insanity. That's enough to excuse – at least legally – the potentially criminal, violent actions of former N.F.L. players in many states.

The Boston University study was published too late to help Aaron Hernandez. He had died – probably because his damaged brain could not reason well enough to prevent him from hanging himself – several weeks earlier.’ (23 September, 2017#1).

This direct link between football, CTE, and violence is assumed to have the potential to cause significant problems for “Football Inc.”. With his diagnosis of CTE, ‘Aaron Hernandez just became the most dangerous man in football’ (*USAT*, 22 September, 2017). It is widely assumed that the NFL's previous attempts to ‘to distance themselves from the disease and the cause-and-effect of playing contact sports’ (*NYP*, 27 September, 2017) is destined to fail in the wake of Hernandez: ‘The NFL is going to own that [Hernandez's death] whether it wants to or not’ (*USAT*, 22 September, 2017). Of note here is that this discussion, in the days after Hernandez's death, seems to differ from the coverage considered by O'Connor and Joffe (2013), which found only ‘superficial’ changes to discussions of responsibility following the insertion of neuroscience into media discourse. While institutional enablement was discussed prior to Hernandez's diagnosis, the tone here is notable in its forcefulness.^3^

Second, there is a narrative which suggests the diagnosis of CTE was inevitable. The diagnosis was ‘hardly surprising’ (*NYP*, 23 September, 2017) and should come as ‘no surprise’ (*NYT*, 23 September, 2017#1; *USAT*, 4 October, 2017) because there was ‘ample evidence’ (*NYT*, 23 September, 2017#1) of CTE when Hernandez was arrested in 2013 and charged with killing Odin Lloyd. As the below quote demonstrates, it is suggested that questions were asked *at the time* of Hernandez's acts:

‘When Aaron Hernandez, the former New England Patriots tight end, was convicted in 2015 of murdering a friend of his, questions arose: Did his years of butting heads on the football field contribute to his violent behavior off the field? And when he killed himself, in April, new questions were asked: Did football play a role in his suicide?’ (*NYT*, 23 September, 2017#2)

While the journalist doesn't specify *where* these questions relating to CTE were asked, and it is entirely possible they were voiced in some other venue, we can say with certainty that they were not being asked within any of the articles sampled here.

A third point of interest concerns Hernandez's “kin”, those drawn close to Hernandez within discourse and deemed to be like him. We have seen that, following his arrest, Hernandez was grouped together with other NFL players who had committed crimes: A kinship which “made sense” because of a shared (raced) culture and the institutional enablement of the NFL, amongst other things. Hernandez and his kin were quite evidently Othered in these narratives: Hernandez was a ‘sociopath’; if not a serial killer then a ruthless ‘gangster’, hanging around with ‘gangbangers’ and adhering to a ‘street code’. Following Rose (2010), we might expect the introduction of a biological narrative to further distance these “monstrous individuals” from the “normal population”. In fact, the opposite appears to occur. Hernandez is still mentioned alongside other NFL players, but no longer only those who died by suicide but also the thousands of others living in fear of CTE (*USAT*, 4 October, 2017). His aggression is also understood in relation to dementia, and ‘uncharacteristic aggression’ is transformed into a trait ‘many caregivers’ can readily relate to (*NYT,* 25 September, 2017#2). Finally, parents and parental decision making is consistently evoked (*NYP* 23 September, 2017; *WP*, 22 September, 2017). Following Hernandez's diagnosis of CTE, ‘parents wonder if they're consigning their kids to a jail cell or the morgue by allowing them to play’ (*USAT*, 22 September, 2017).

On the basis of this reporting, it seems plausible that there are two factors that, contra the analyses of Rose, bring Hernandez into closer contact with the “normal” population. First, the ‘clinical and pathological parallels’ (*NYT*, 25 September, 2017#2) with other forms of dementia. If CTE remains an exceptionally rare diagnosis, dementias in general are not and, furthermore, the ‘dominant perceptions of the ‘disease’ [dementia] are framed primarily around *loss* of self’ (Swallow, 2017, p. 59, emphasis added). Thus, by entangling dementia and aggression, the suggestion is raised that violence may not have been an inalienable part of Hernandez's subject or a result of his “culture” but, rather, a consequence of neurodegeneration: readers are asked to identify with this rendering through the surrogate figure of the dementia patient they care for. Second, Hernandez's brain injury, which in this sample is strongly implicated in his actions and associated with football, was *acquired* and it was acquired through participation in a sport which a great many law-abiding athletes – not to mention journalists, readers, and their children – also participate or have participated in. Thus, the responsibility of Hernandez is questioned at the same moment that parents and custodians become implicated in the story: parents are “responsiblized” (cf: Biebricher, 2011; Rose and Novas, 2005) and asked to confront the possibility that they may, in fact, *make* their children Hernandez's kin. It is a limited sample size but I tentatively suggest that it is in this space – between the acquired nature of CTE and the violence and loss of self associated with dementia – that the present discourse is shaped.

## 5 Conclusion

Following Aaron Hernandez's suicide, an article in *USAT* (20 April, 2017 #1) described the case as ‘a true American horror story’, ‘a grim American tale’, and an ‘American tragedy’. James Patterson, one of the world's bestselling authors, has written a book about Hernandez entitled *All-American Murder* (2018). If it is true that Hernandez's story of sporting glory, celebrity, violence, and finally suicide speaks profoundly to the contemporary American condition, what role does medicine/neurology play?

Numerous scholars (e.g. Dumit, 2004; Novas and Rose, 2000; Rabinow, 1999) have argued that recent decades have seen a biologicalization of selfhood as individuals use genetic and neurological terms in their practices of self-fashioning. Research has articulated an apparent medicalization of concussion (Hardes, 2017; Malcolm, 2016) and a “cultural awakening” over CTE (Anderson and Kian, 2012; Ventresca, 2019) in these terms.

My examination of the media discourses surrounding the arrest, conviction, suicide, and diagnosis of Aaron Hernandez has only partially supported this thesis. It is certainly true that, following a posthumous diagnosis of CTE, understandings of Hernandez's behaviour change significantly: his culpability (for both his criminal behaviour and his suicide) is questioned and diminished; his aggression is no longer unquestioningly monstrous and is brought into contact with the lived-experience of caregivers supporting individuals with dementia. CTE, undoubtedly, became entangled with the story of Aaron Hernandez.

Nonetheless, this intertwining of sporting subjectivity and neurological state was highly dependent upon a *specific moment of diagnosis*. Despite occurring in a “post-Webster era” when discussion of CTE is, apparently, to the fore, there was no mention of Hernandez's brain or the possibility of neurodegenerative disease during coverage of either his arrests or subsequent conviction. Even Hernandez's suicide and the removal of his brain for diagnostic testing brought little discussion of CTE. Instead, family instability, ‘hanging with the wrong crowd’ (which was frequently racialized), individual psychology, and the entitlement of celebrity aided and abetted by Football Inc. were all found to be *general* explanations more readily deployed in the media in order to understand Hernandez's behaviour.

This last finding brings a particular valence to the discussion: Hernandez was only interpellated into the discourse of CTE (by the media at least) once he was diagnosed, and diagnosis is *only possible* post-mortem. This situation, quite evidently, is very different to that found in depression, schizophrenia, personality disorder, or the vast majority of other conditions considered within biologicalization literature. We are thus reminded that the entanglement of bio/neuroscience with selfhood ‘ … may depend, to some extent, on the specific characteristics of the condition in question’ (Weiner, 2011, p. 1766). Quite why it took the sampled media so long to consider Hernandez ‘under the description’ (Martin, 2007, p. 10) of CTE – especially given various assertions after-the-fact that a diagnosis was predictable or even inevitable – and quite what the consequences are of interpellation occurring only after death require further studies and alternative methods. Nonetheless, the matter seems important ethically, legally, and sociologically and is worthy of further attention.

## Footnotes

1 Different databases were used as outlets were not available via one source. A full list of newspaper articles consulted in this analysis is available from https://hardknocklife.hcommons.org/downloads/.

2 The exception to this is the murder of Odin Lloyd as Hernandez was arrested one week after the murder.

3 The newspaper articles published in the two weeks after Hernandez's diagnosis are, largely, univocal on this point: They stress some uncertainty while simultaneously drawing a more-or-less straight line between Hernandez's brain and his behaviour. The broader media narrative, however, is less straightforwardly linear. For example, and while outside the scope of this article, various long form pieces produced about Hernandez (e.g. Baez, 2018; Hohler, 2018; Hernandez and Andersen, 2018; Patterson et al., 2018) have drawn attention to a multiplicity of factors when attempting to understand his actions.
